# Removal of sacral neuromodulation quadripolar tined-lead using a straight stylet: description of a surgical technique

**DOI:** 10.1007/s10151-020-02403-6

**Published:** 2021-04-22

**Authors:** M. Agnello, M. Vottero, P. Bertapelle

**Affiliations:** 1 Scuola di Medicina, Dipartimento di Scienze Chirurgiche, SC Neuro-Urologia, A.O.U. Città della Salute e della Scienza di Torino, Università degli Studi di Torino, Turin, Italy; 2 SC Neuro-Urologia, A.O.U. Città della Salute e della Scienza, Turin, Italy

**Keywords:** Sacral neuromodulation, Quadripolar tined-lead, Lead removal, Technique, Stylet, Straight stylet

## Abstract

**Background:**

Up to 7.5% of tined-lead removals in patients having sacral neuromodulation (SNM) therapy are associated with a lead breakage. It is still unclear what adverse effects can be caused by unretrieved fragments. The aim of our study was to describe the lead removal technique we have been using for the last 2 years in our centre.

**Methods:**

We retrospectively enrolled patients who had lead removal between January 2018 and January 2020 using our standardized technique. The novelty of the technique is in the use of the straight stylet, which is available in the quadripolar tined-lead kit. The stylet gives the electrode greater stiffness, reducing interactions with surrounding tissues and probability of damage or breakage during removal.

**Results:**

In 59 patients (42 women, mean age 57.2 years [range 40–79 years]) the lead was removed using our standardized technique. In 44 of 59 patients, the tined-lead was removed within 2 months from the SNM-test, due to lack of beneficial effects. In 15 patients the electrode was removed because of failure of definitive implantation. Meantime from definitive implantable pulse generator (IPG) implantation to lead removal was 67.9 months. We recorded only 1 case of lead-breakage during removal: a female patient with a non-tined lead fixed on sacral bone, placed 18 years previously using an open technique.

**Conclusions:**

Lead breakage during removal is not uncommon and adverse effects of retained fragments may occur. Our technique has been safely used for the last 2 years in our centre, with no episodes of lead breakage or retained fragments, except for one non-tined electrode.

**Supplementary Information:**

The online version contains supplementary material available at 10.1007/s10151-020-02403-6.

## Introduction

Sacral neuromodulation (SNM) is typically a two-step procedure. During the SNM-test, a quadripolar tined-lead is placed in the third sacral foramen using a standardized percutaneous electrode-placement technique [1].

Normally, if follow-up is positive and clinically significant benefits from nerve stimulation are observed, the temporary extension is removed and a definitive implantable pulse generator (IPG) is placed in a gluteal pocket. In the absence of clinical improvement, the temporary extension and quadripolar tined-lead are removed. Based on clinical and functional observations, a contralateral test or a replacing of the lead may be required.

In many cases, SMN has to be performed several times, and testing electrodes often need to be removed and occasionally replaced.

**Fig. 1:**
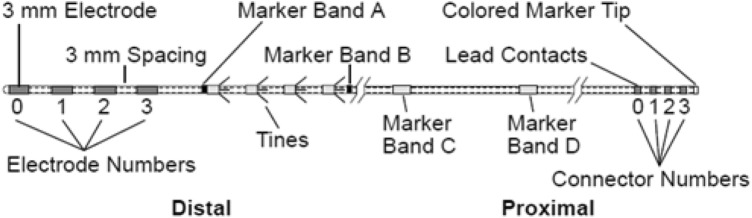
Model 3889 lead (adapted from “Medtronic Manuals: InterStim® Therapy Model 3093 Lead Model 3889 lead—implant manual [17]”). Tines lie between marker band A and marker band B

The currently available quadripolar lead incorporates four equally spaced contact points and four tines (fins), which keep the lead in place once it has been inserted in the sacral foramen (Fig. 1). The position and orientation of tines on the lead make it easy to introduce the electrode during the first-step procedure, but harder to remove it by pulling outward. This mechanism has been studied to avoid accidental lead displacement after implantation [2]. Before the introduction of the percutaneous technique, open surgery was necessary to reach the third sacral foramen and the lead was surgically fixed to periosteum.

Approximately 20–30% of SNM-implanted patients require surgical revision, whilst 15–18% of them have definitive explantation [3–11]. It is estimated that, regardless of the indication for the procedure, quadripolar lead removal carries a 7.5% risk of lead breakage and permanence of fragments within the pelvis or proximal to the sacral bones [12].

The aim of our study was to describe the technique we have been using for the last 2 years in our centre for the removal of the quadripolar lead both after SNM-testing and following complications of definitive IPG implantation.

## Materials and methods

We searched the SNM database of our institution for all the patients who had a quadripolar lead removal between January 2018 and January 2020 via our standardized technique. The study was conducted according to the Declaration of Helsinki general principles for medical research involving humans. Following our Institutional Ethical Committee Approval (protocol no. 0036753) and acquisition of written informed consent, patient data were collected. The technique is described below. A step-by-step video is attached.

### Patient positioning and preparation

The patient is placed in a prone position with the hips supported to minimise lumbar lordosis and with all pressure points adequately padded. If a temporary extension is present, it is secured with self-retaining surgical forceps.

### Local anaesthesia, skin incision and device isolation

A local anaesthetic (such as lidocaine 1%, ropivacaine 7.5%) is used to infiltrate scars of previous percutaneous lead insertion and the gluteal pocket. The skin is incised on the gluteal pocket. Subcutaneous tissues are gently dissected to avoid lead damage, preferably using a scalpel rather than monopolar current. Lead integrity is of crucial importance to introduce the stylet. If an IPG is present, the pseudocapsule is opened and the device is disconnected from the quadripolar lead. If a SNM-test was performed, the connector of percutaneous extension is identified, isolated and sectioned.

### Quadripolar lead isolation

**Fig. 2:**
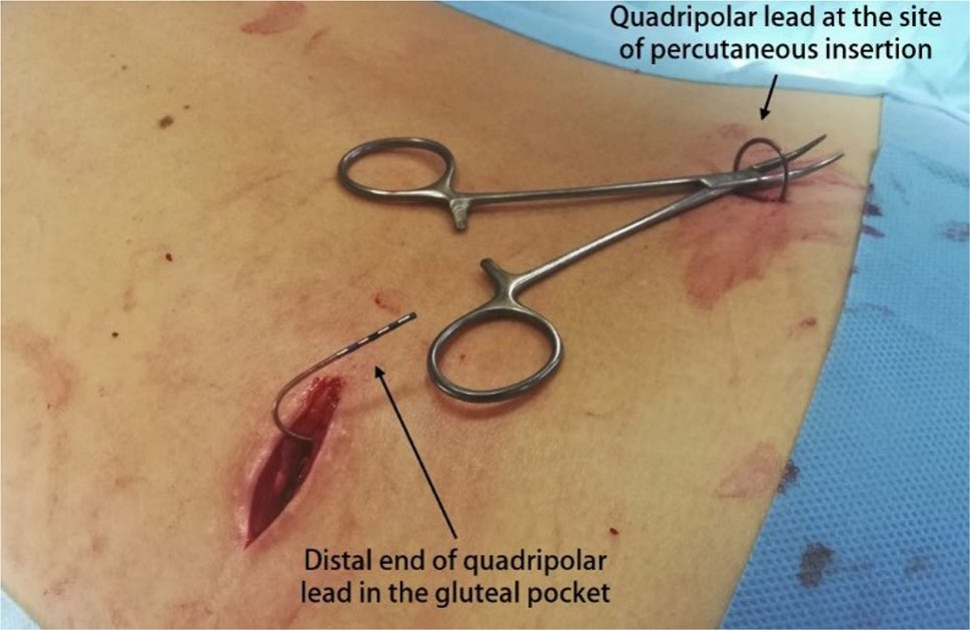
Isolation of quadripolar lead from the gluteal pocket and the sacral incision

Once the distal end of the quadripolar lead is freed from the IPG or the external connector, a gentle traction on the electrode allows identification of a small skin depression over the site of the percutaneous implant. This manoeuvre is useful to assess if the electrode is well isolated and mobile from surrounding tissues, or requires further dissection within the gluteal pocket. A skin incision is made over the sacral scar, and the electrode is isolated as it enters the S3 foramen. Care should be taken to avoid damaging the wire. It is then possible to pull the electrode and extract it through the small sacral incision (Fig. 2).

### Use of the stylet to straighten the electrode

**Fig. 3:**
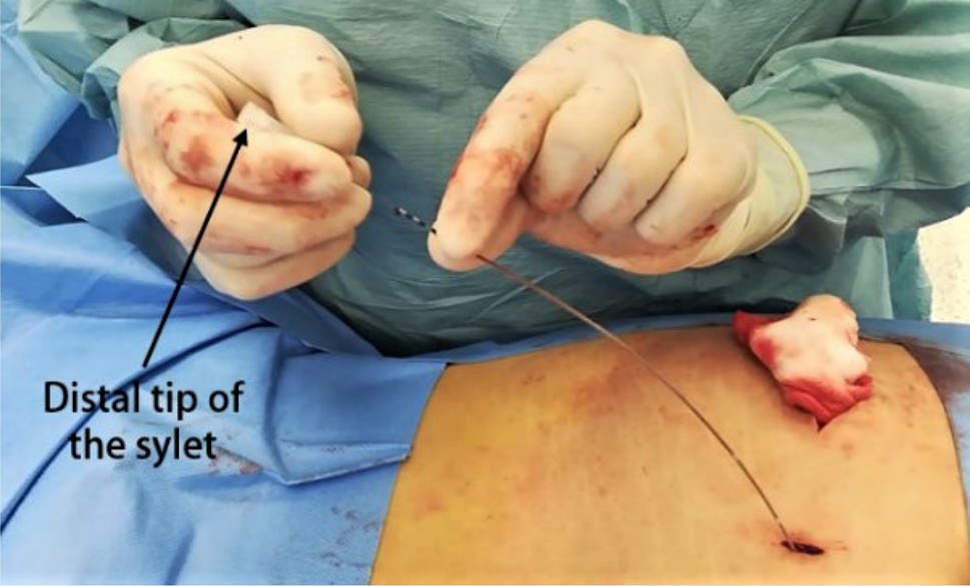
Introduction of the stylet inside the electrode, to straighten it

**Fig. 4:**
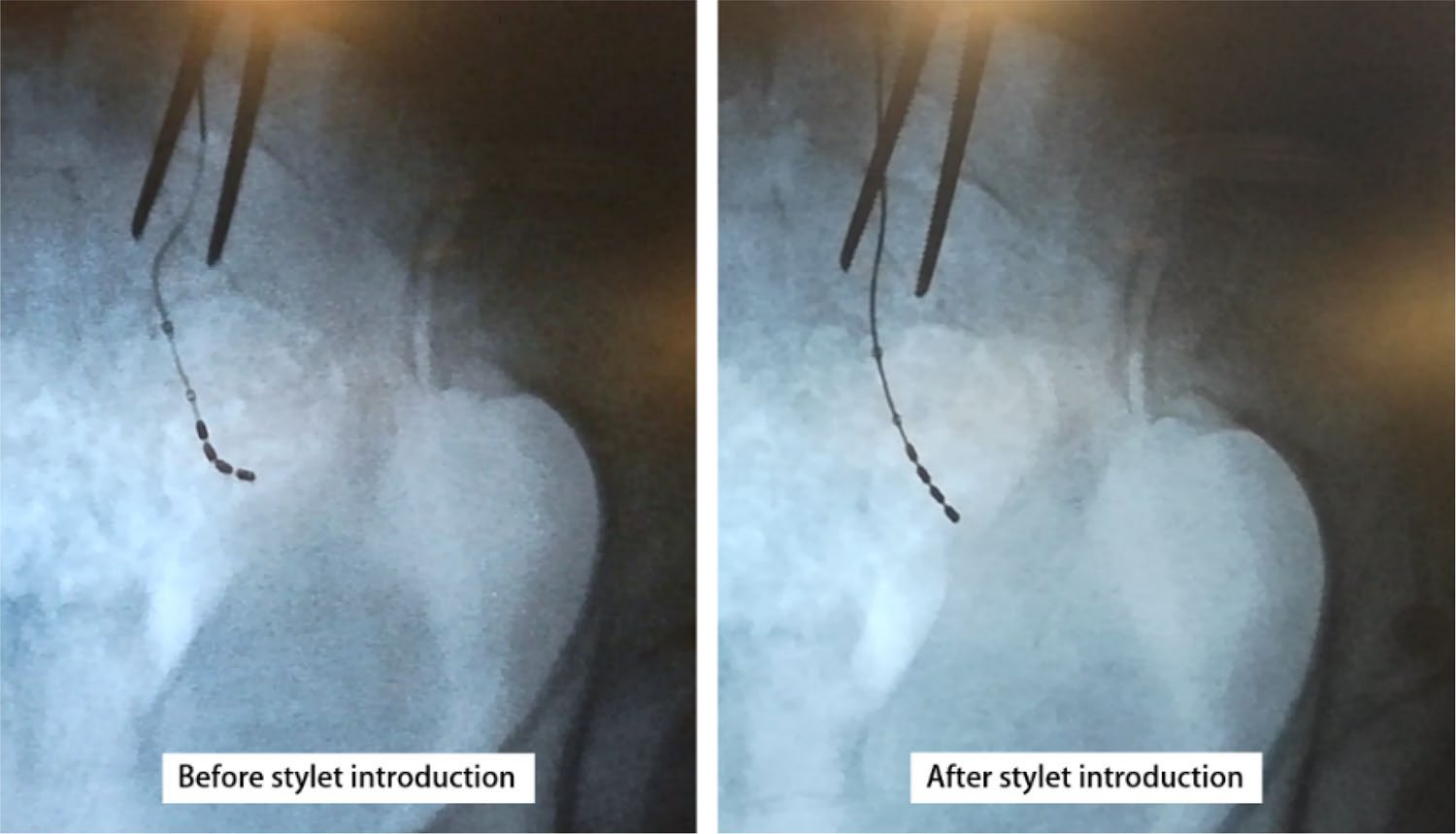
X-ray imaging during the lead removal procedure. It is possible to see the effect of using the stylet in straightening the electrode. The use of X-ray imaging is not part of the procedure. It was performed in the first patients to verify the manoeuvre and is here reported for a clinical purpose

Within the original packaging two stylets are provided, one straight and one curved. They are sterilized by the manufacturer using ethylene-oxide (EtO sterilization process). At the time of the percutaneous implant, the surgeon selects which stylet to use [1]. In our experience, we tend to use the bent stylet for the SNM-test, and store the straight one after a second sterilization process with saturated steam (autoclave, temperature 134°, pressure 2.1 bar, 1 h for a complete cycle). The straight stylet is kept for a maximum of 1 month and can be used for a lead removal procedure in this period. In our technique, the surgeon places the straight stylet inside the electrode through its distal end (Fig. 3). In this way, straightening of the whole lead is obtained, including its proximal end in the pelvis, which is typically curved and in a caudo-lateral direction. In the first 3 cases of our series, we performed X-ray imaging during the procedure to confirm straightening of the electrode inside the pelvis (Fig. 4). However, X-ray was done exclusively for specific clinical purposes and is not routinely recommended.

### Quadripolar tined-lead removal

Once the stylet is inserted, the first assistant uses a clamp on the electrode to keep it in place. Subsequently, the subcutaneous tissue is gently dissected until the electrode marker band B, at the apex of tined-region, is exposed. A second clamp is used to grip the electrode under the marker band. A torsional movement with soft traction of the clamp will allow the fascia to separate from the tines without breaking the electrode, which can now be easily removed. The stiff stylet straightens the electrode and renders it more rigid, reducing the risk of electrode breakage.

## Results

We retrospectively enrolled 59 patients (41 women and 18 men) who had quadripolar lead removal using our standardized technique between January 2018 and January 2020. Mean patient age was 57.2 years, and mean patient body mass index (BMI) was 22.5.kg/m^2^.

In 44 patients, a tined-lead was removed within 2 months from the SNM-test, due to the absence of clinically significant benefits. In 15 patients the electrode was removed after definitive IPG implantation. In this cohort, mean time from definitive IPG implantation to lead removal was 67.9 months. The electrode was permanently removed in one-third of patients, whilst it was replaced in the remaining cases. Patient characteristics and indications for lead implant and removal are shown in Table 1.

**Table 1:** The patients who had lead removal after definitive IPG implantation

Patient ID	Sex	Year of birth	Date of lead implant	Date of lead removal	Time from implant to removal (months)	Implant reason	Reason for lead removal	Lead substitution	Lead breakage
1	F	01/01/67	07/11/18	02/05/19	5	Urinary retention	Loss of efficacy	No	No
2	F	01/01/50	14/09/16	28/03/18	18	Overactive bladder syndrome	Loss of efficacy	No	No
3	F	01/01/72	18/01/17	01/08/18	18	Urinary retention	Sub-optimal lead position at X-ray (deep in the pelvis) and partial loss of efficacy	Yes	No
4	F	01/01/79	12/04/17	15/05/19	25	Urinary retention	Pain over the site of percutaneous implant due to a sudden weight loss	Yes	No
5	F	01/01/65	07/09/16	08/05/19	32	Interstitial cystitis	Sub-optimal lead position at X-ray (sideway to the arch of S3 foramen) and partial loss of efficacy	Yes	No
6	F	01/01/67	01/12/15	07/11/18	35	Urinary retention	Sub-optimal lead position at X-ray (deep in the pelvis) and partial loss of efficacy	Yes	No
7	F	01/01/56	15/12/14	20/06/18	42	Urinary retention	Loss of efficacy	No	No
8	F	01/01/69	15/04/15	02/05/19	48	Urinary retention	Need for MRI for other clinical reasons, and partial loss of efficacy	No	No
9	F	01/01/54	06/11/13	31/05/18	54	Overactive bladder syndrome	Device malfunctioning with high impedance	Yes	No
10	F	01/01/45	17/06/10	31/01/18	91	Urinary retention	Loss of efficacy	Yes	No
11	F	01/01/39	14/04/10	31/01/18	93	Faecal incontinence	Sub-optimal lead position at X-ray and CT scan (S4) and partial loss of efficacy	Yes	No
12	F	01/01/45	24/03/10	24/01/18	94	Overactive bladder syndrome	Loss of efficacy	No	No
13	F	01/01/64	03/09/08	24/01/18	112	Urinary retention	Substitution of IPG 3023 model with an IPG 3058 model	Yes	No
14	M	01/01/42	18/04/07	14/03/18	130	Urinary retention	Loss of efficacy	Yes	No
15	F	01/01/44	01/01/01	12/06/19	221	Overactive bladder syndrome	Sub-optimal lead position at X-ray and CT scan (S4) and partial loss of efficacy	Yes	*Yes*

Patients are sorted by the time between IPG definitive implant and lead removal (ascending order). We experienced a lead breakage in only one patient (Patient ID 15). It was a not-tined lead placed 18 years before with fixation to sacral periosteum through an open surgery. Two-thirds of patients (10/15) replaced the electrode concurrently to the removal procedure. In the remaining cases, the lead was permanently removed, mainly due to a loss of efficacy

*MRI* magnetic resonance imaging, *CT scan* computed tomography scan, *IPG* implantable pulse generator

We recorded only 1 case of lead breakage during lead removal, which occurred in a female with a not-tined lead placed 18 years before (221 months), through an open technique with a surgical fixation on the periosteum of the sacral bone. The lead was placed in the fourth sacral foramen, and the device lost its efficacy progressively over the few years prior to the removal. Lead removal by gentle traction was not possible, and it was decided not to proceed with a deeper surgical extraction. A new quadripolar tined-lead was placed in the S3 foramen on the same side, using the percutaneous technique.

## Discussion

Rueb et al. [12] found a 7.5% breakage rate during the quadripolar lead removal procedure and identified the tined region as the most vulnerable to damage. The consequences of non-removal of the fragments remain unclear. The fragments increase the risk of motion artefact, involuntary stimulation or heating of the lead during magnetic resonance imaging [13, 14] and other radiological investigations. Other complications include pain at the site of fragments [12] and technical and surgical difficulties in case of new percutaneous lead placement procedures. Lead fragment migration into the sigmoid colon and a subsequent colocutaneous fistula has also been described [15].

In 2008 the United States Food and Drug Administration (FDA) warned clinicians to take specials precautions to prevent devices from breaking and being left in patients, due to potentially serious adverse events related to unretrieved device fragments (UDFs) [16]. The Medtronic implant manual of lead model 3093 and 3889 merely recommends the application of gentle traction on the electrode, while removing it through the lead introducer site, in a straight line from the lead tines. If resistance occurs during lead removal, a deeper dissection may be necessary to avoid lead breakage and UDFs [17]. The International Continence Society endorses these recommendations [18].

To our knowledge, the most accepted and the only standardized technique for lead removal is the one described by Sterling et al. [19] (and by Okhunov et al. [20] shortly after), which consists of isolating and externalizing the lead with a small skin incision on the previous lead insertion site, dissecting the surrounding fibrous tissue to expose the tines, and removing the lead by wrapping it around a curved hemostat and turning it under tension. Using this technique, and possibly extending the incision down to the sacral body if fragments are present, only 1 lead (3.6%) broke in Sterling’s series.

Two other non-standardized techniques are occasionally reported: lead removal through an incision over the IPG site and gentle lead traction from the gluteal pocket, and the use of the straight stylet to help straighten the electrode.

All these studies focused on the removal of the quadripolar lead after definitive IPG implantation when formed adhesions around tines could make it harder to remove the lead, and description of a specific removal technique is justified [12, 19, 20]. There is no doubt that the more time passes from lead insertion, the more challenging it is to remove it. However, adhesions around the lead due to inflammation may also occur after stage 1 and during the SNM-testing period. Furthermore, the electrode may migrate deep into the pelvis shortly after its insertion and might need to be safely removed. Whenever fragments of tined lead, especially stimulating poles, remain in place, they prevent new electrodes from being implanted at the same site. A limitation of our study is that only 15 patients had the lead removed after definitive IPG implantation. In the remaining 44 cases, the lead has been removed after the 2-months testing-period during which most authors agree that it is easier to avoid lead breakage.

We experienced only 1 lead breakage during the removal procedure after a definitive IPG implantation. However, the patient was the only case out of the series where the open technique—with non-tined lead fixation to the sacral bone—had been used instead of the typical percutaneous insertion. In such cases, we know that the only way to completely remove the lead consists of dissecting deep tissues and removing the plate and the screws used for fixation.

Strengths of our technique include the ease of application and the use of an instrument (the stylet) that is already provided inside the original Medtronic package. Furthermore, the introduction of the stylet does not increase the operative times nor the peri-operative risks for the patient and does not substantially alter the techniques previously described. The benefit of straightening the electrode by reducing interactions with the surrounding tissue during removal significantly outweighs the risks associated with the procedure.

## Conclusions

Our technique using a straight stylet to remove the electrode, has been safely used for the last 2 years in our centre, with no episodes of tined electrode breakage or retained fragments.

## Supplementary Information

Below is the link to the electronic supplementary material.

Supplementary file 1 (MP4 304596 KB)
